# Risk Factors for Liver Decompensation and HCC in HCV-Cirrhotic Patients after DAAs: A Multicenter Prospective Study

**DOI:** 10.3390/cancers13153810

**Published:** 2021-07-29

**Authors:** Filomena Morisco, Alessandro Federico, Massimo Marignani, Mariarita Cannavò, Giuseppina Pontillo, Maria Guarino, Marcello Dallio, Paola Begini, Rosa G. Benigno, Flavia L. Lombardo, Tommaso Stroffolini

**Affiliations:** 1 Gastroenterology and Hepatology Unit, Department of Clinical Medicine and Surgery, University of Naples “Federico II”, 80131 Naples, Italy; filomena.morisco@unina.it (F.M.); giu.pontillo@gmail.com (G.P.); 2 Hepato-Gastroenterology Unit, University of Campania Luigi Vanvitelli, 80131 Naples, Italy; alessandro.federico@unicampania.it (A.F.); marcello.dallio@gmail.com (M.D.); 3 Department of Liver Diseases Section, AOU Sant’Andrea Hospital, University of Hospital La Sapienza, 00189 Rome, Italy; massimo.marignani@uniroma1.it (M.M.); paolabegini@virgilio.it (P.B.); 4 Liver Unit, Department of Internal Medicine, ARNAS Garibaldi, 95100 Catania, Italy; mariaritac@alice.it (M.C.); r.benigno@ao-garibaldi.ct.it (R.G.B.); 5 National Center for Disease Prevention and Health Promotion, Italian National Institute of Health, 00161 Rome, Italy; flavia.lombardo@iss.it; 6 Department of Tropical and Infectious Disease, Policlinico Umberto I, 00161 Rome, Italy; tommaso.stroffolini@hotmail.it

**Keywords:** DAAs, HCV, HCC, liver decompensation, liver stiffness, cirrhosis

## Abstract

**Simple Summary:**

The present study explored the predictors of the development of liver-related events in HCV cirrhotic subjects achieving SVR following antiviral therapy with direct-acting antiviral agents (DAAs) during a follow-up of 24 months after SVR confirmation. Patients had a liver stiffness measurement (LSM) of ≥14 kPa at baseline. We found that baseline liver stiffness ≥ 20 kPa and HCV genotype different from 1 were both independent predictors of liver decompensation, while only LSM ≥ 20 kPa was an independent predictor of HCC.

**Abstract:**

Background: Prospective studies on predictors of liver-related events in cirrhotic subjects achieving SVR after DAAs are lacking. Methods: We prospectively enrolled HCV cirrhotic patients in four Italian centers between November 2015 and October 2017. SVR and no-SVR cases were compared according to the presence or absence of liver-related events during a 24-month follow-up. Independent predictors of liver-related events were evaluated by Cox regression analysis. Results: A total of 706 subjects started DAAs therapy. SVR was confirmed in 687 (97.3%). A total of 61 subjects (8.9%) in the SVR group and 5 (26.3%) in the no-SVR group had liver-related events (*p* < 0.03). The incidence rate x 100 p/y was 1.6 for HCC, 1.7 for any liver decompensation, and 0.5 for hepatic death. Baseline liver stiffness (LSM) ≥ 20 kPa (HR 4.0; 95% CI 1.1–14.1) and genotype different from 1 (HR 7.5; 95% CI 2.1–27.3) were both independent predictors of liver decompensation. Baseline LSM > 20 KPa (HR 7.2; 95% CI 1.9–26.7) was the sole independent predictor of HCC. A decrease in liver stiffness (Delta LSM) by at least 20% at the end of follow-up was not associated with a decreased risk of liver-related events. Conclusion: Baseline LSM ≥ 20 kPa identifies HCV cirrhotic subjects at higher risk of liver-related events after SVR.

## 1. Introduction

Experience with new direct-acting antiviral agents (DAAs) treatment for chronic HCV infection has shown that more than 95% of treated subjects, even those with liver cirrhosis, achieves sustained virological response (SVR), i.e., clearance of the virus [1]. This rate of cure is much higher than the corresponding rate of <20% obtained in cirrhotic patients previously treated with Interferon-based (IFN) therapies [2], generating a great increase in subjects cured of HCV infection.

Several studies compared SVR versus no-SVR cases to assess the rate of independent predictors for liver-related events. Cirrhotic subjects who achieved SVR after DAAs treatment had significantly lower rates of liver-related events, including de novo HCC development, as compared to those who did not achieve SVR [3,4,5,6,7,8].

Nonetheless, cirrhotic subjects who achieved SVR continued to develop liver-related events, albeit at a lower rate [9]. There is limited information on predictors of unfavorable outcomes after successful viral eradication.

Two US retrospective studies [10,11] and one Italian retrospective study [12] focused only on HCC occurrence. Only a Spanish prospective study [13] evaluated predictors of liver events related to portal hypertension (ascites, variceal bleeding, hepatorenal syndrome, hepatic encephalopathy) and HCC in the subgroup of “compensated advanced chronic liver disease” (cACLD), a population free of previous decompensation events but characterized by the presence of signs of portal hypertension [14]. This population is of obvious interest, but it does not represent all cirrhotic cases.

To further expand the currently limited information on this issue, we evaluated the non-serological baseline predictors of liver-related events’ development in cirrhotic subjects (regardless of Child–Pugh stage) achieving SVR after DAAs therapy in a prospective manner.

## 2. Results

### 2.1. Overall Cohort

**Figure 1:**
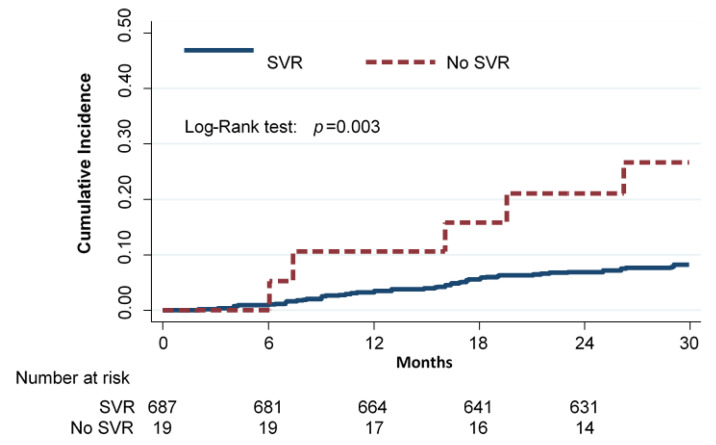
Cumulative incidence of any event (deaths for hepatic and non-hepatic cause, liver transplantation, liver decompensation, and HCC) in cirrhotic subjects according to SVR (Kaplan–Meier estimates).

A total of 706 HCV-RNA-positive cirrhotic patients started DAAs therapy. SVR was confirmed in 687 (97.3%) of them. A total of 66 subjects had at least one liver-related event during a mean follow-up of 28.4 ± 5 months: 61 (8.9%) in the SVR group and 5 (26.3%) in the no-SVR group. Ten subjects (nine in the SVR group; median follow-up 1.25 years, range 0.7–1.5) were lost to follow-up; none of them, however, reported liver-related events up to the last observation performed. The Kaplan–Meier cumulative incidence curves of any event by SVR status are shown in Figure 1 (*p* < 0.003 by log-rank test).

The incidence rate of any event (i.e., liver-decompensation, HCC, and death for any cause) per 100 person-years was 3.7 (95% CI 2.9–4.8) in the SVR group and 12.3 (95% CI 5.1–29.6) in the no-SVR group. The relative rate (RR) of any event for subjects who did not achieve SVR compared to subjects who achieved SVR was 3.3 (95% CI 1.3–8.2).

In SVR patients, eight liver-related deaths were recorded, and two were recorded in no-SVR patients. The incidence rate per 100 person-years was 0.5 (95% CI 0.2–1.0) in the SVR group and 4.3 (95% CI 1.1–17.2) in the no-SVR group. The RR or incidence rate ratio for subjects who did not achieve SVR compared to subjects who achieved SVR was 9.0 (95% CI 1.9–42.6). See Table S1A.

### 2.2. SVR Cohort

The baseline characteristics of the 687 subjects who achieved SVR are shown in Table 1. The male/female ratio was 1.2; the mean age was 64 years. More than half of them (53.4%) reported previous IFN therapy. Genotype 1 was predominant (80.1% of cases). As many as 92.7% of cases had Child–Pugh A stage cirrhosis, 7.3% Child–Pugh B, and none Child–Pugh C. Liver stiffness was measured in 567 (82.5%) subjects; most of them (70.6%) had a basal value < 20 kPa.

In total, 15.4% (106/687) of SVR subjects had an improvement in the Child–Pugh score at the last evaluation in comparison to the baseline value. In particular, 72.7% of Child–Pugh B patients had an improvement in liver function, obtaining a Child–Pugh score of A after SVR.

### 2.3. Occurrence of Events in SVR Cohort

The incidence rate × 100 person-years was 1.2 for overall death (0.5 for hepatic and 0.7 for not-hepatic cause), 1.7 for liver decompensation, 1.6 for HCC, and 0.1 for liver transplantation. The overall incidence of any event was 3.7 × 100 person-years (Table 2).

### 2.4. Decrease in Liver Stiffness Measurement (Delta LSM)

At the end of follow-up, liver stiffness was measured in 490 (86.4%) of the 567 subjects who had a liver stiffness measurement at the beginning of the study. Of them, 258 (52.6%) had a ≥20% decrease in liver stiffness value as compared to baseline. The only baseline factor associated with a liver stiffness decrease of ≥20% was a genotype different from 1 (*p* < 0.04) (Table 3). Comparing subjects with Delta LSM of ≥ or <20% of the basal value, those below this cut-off did not appear to be at a higher risk of liver decompensation (incidence rate 0.6 vs. 0.5; RR 1.2, 95% CI = 0.3–5.4) or of HCC occurrence (incidence rate 0.8 vs. 0.3; RR 2.3, 95% CI = 0.4–11.8). See Table S1B.

### 2.5. Basal Predictors of Liver-Related Events

**Figure 2:**
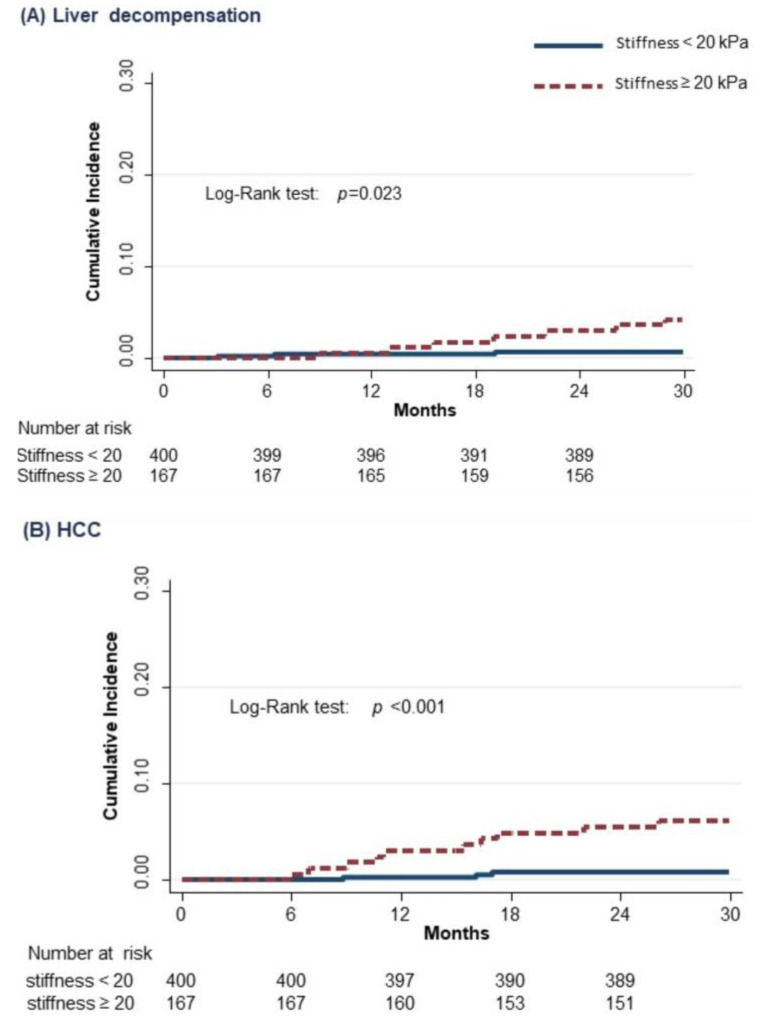
Cumulative incidence of liver decompensation (**A**) and HCC (**B**) in cirrhotic subjects according to the value of stiffness at baseline (Kaplan–Meier estimates).

**Figure 3:**
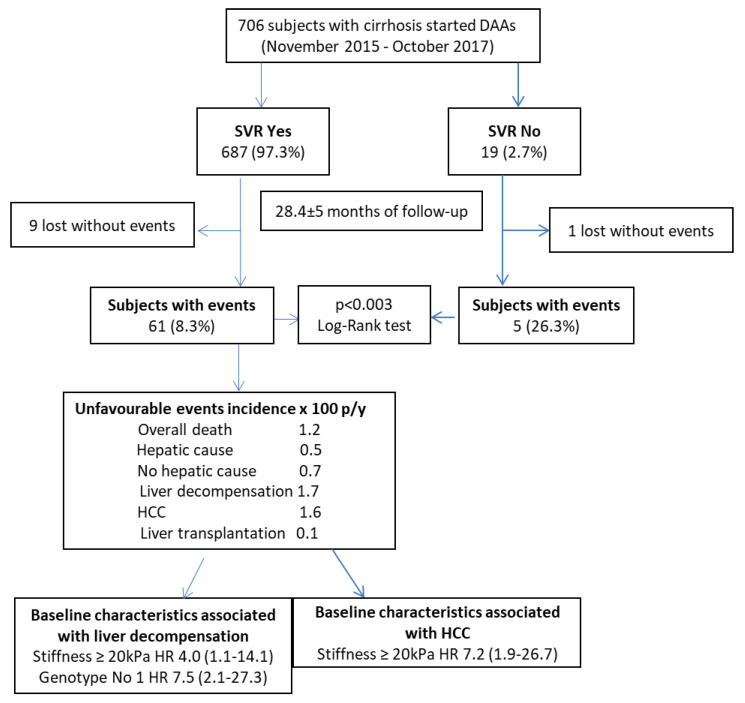
Study flowchart with main results.

The crude and adjusted HR for the association of non-serological baseline characteristics of SVR subjects with liver-related events are shown in Table 4. Liver stiffness ≥20 kPa (HR 4.0; 95% CI 1.1–14.1) and a genotype different from 1 (HR 7.5; 95% CI 2.1–27.3) were both independent predictors of liver decompensation, while liver stiffness ≥20 kPa (HR 7.2; 95% CI 1.9–26.7) was the only independent predictor of HCC development. After the analyses for competing risks (i.e., considering death for any cause of a competing event), the association of liver-related events with the considered variables did not change. The cumulative incidences of liver decompensation and HCC according to the value of liver stiffness at baseline are shown in Figure 2. The incidence rate of liver decompensation per 100 person-years was 0.4 (95% CI 0.1–1.08) in subjects with basal LSM value < 20 kPa, as compared to 2.2 (95% CI 1.2–4.3) in those above this cut-off (RR = 5.5) (95% CI = 1.7–17.8) (Figure 2A); the incidence rate of HCC was 0.3 (95% CI 0.1–0.9) in subjects with basal LSM value < 20 kPa, as compared to 2.5 (95% CI 1.4–4.7) in those above this cut-off (RR = 8.3) (95% CI 2.3–30.2) (Figure 2B). Finally, Figure 3 summarizes the study flowchart with the main results.

## 3. Discussion

Until now, the largest published cohort of cirrhotic patients treated with DAAs outside a liver transplant setting is the study reported by Cheung et al. [15], which analyzed 406 patients, most of them Child–Pugh B (41 patients were Child–Pugh C), and followed-up for 15 months. The majority of patients had a reversal of liver decompensation after DAAs therapy, with no evidence of an increased risk of liver malignancy. Our study proposes instead, with a prospective design, to evaluate the predictors of any liver-related event (decompensation and HCC) in cirrhotic subjects in Child–Pugh stage A and B achieving SVR after oral antiviral therapy with follow-up extended to 24 months for all subjects.

The main finding of the present study is the role of baseline liver stiffness (≥20 kPa) as an independent predictor of liver-related event development. Compared to subjects with baseline liver stiffness <20 kPa, those who had an LSM above this cut-off showed a four-fold higher risk of liver decompensation and a seven-fold higher risk of HCC after adjustment for the confounding effect of baseline variables by the Cox regression model.

Liver stiffness measurement by transient elastography is an accurate indirect, non-invasive method to assess liver fibrosis grade [16], a strong risk factor for HCC and for portal hypertension. In fact, LSM has been shown to have good accuracy in predicting the presence of cirrhosis [17] and clinically significant portal hypertension [18,19,20], as confirmed by the Baveno VI consensus document [14].

An increased basal LSM value, evaluated as a continuous variable, has been associated with an increased risk of HCC in several studies [12,21,22,23,24,25,26]. In a Japanese study, when compared to basal LSM ≤ 10 kPa, the adjusted hazard ratio for HCC development result was 16.7 when LSM was between 10.1 kPa and 15 kPa, 20.9 when LSM was between 15.1 kPa and 20 kPa, 25.6 when LSM was between 20.1 kPa and 25 kPa, and finally, the result was 45.5 when LSM was >25 kPa after adjustment for other significant factors for HCC [21].

The cut-off of 20 kPa identified in the present study is in line with the data produced by Lens et al., who identified a cut-off of 21 kPa to separate patients with and without benefit from SVR in the presence of clinically significant portal hypertension [27].

In agreement with our findings, a previous retrospective Italian study [12] using a higher basal LSM cut-off (≥30 kPa) showed an increased, although marginally associated, risk of HCC occurrence (HR 1.03; 95% CI = 1.01–1.06) during a median follow-up of 24 months. Conversely, a Spanish study [13] identifying a similar basal LSM cut-off (≥20 kPa) failed to show an increased risk of HCC occurrence. This discrepancy may be explained by a selection bias: the Spanish study recruited only a subset of cirrhotic subjects, i.e., those with well-compensated disease, while our survey enrolled even cirrhotic patients with more advanced disease (i.e., Child–Pugh A with prior decompensation and Child–Pugh B).

Change in liver stiffness value over time (Delta LMS) has been proposed as an accurate and reliable predictor of liver decompensation [28] and HCC [29]. Unfortunately, in both this study and the Spanish study [13], a ≥20% decrease in Delta liver stiffness at the end of follow-up was not associated with a reduced risk of HCC and liver decompensation. The relative early change of LSM probably reflects a greater improvement in liver inflammation than in fibrosis, which may require longer follow-up to be observed.

Compared to Child–Pugh A cases, the enrolled Child–Pugh B cases did not show a higher risk of subsequent liver decompensation or HCC. Additionally, most subjects showed an improvement in liver function, becoming Child–Pugh A with a subsequent decreased risk of liver-related events. Conversely, a recent retrospective study has shown that in patients with chronic HCV infection, DAAs-induced SVR was associated with a reduced risk of liver-related events in Child–Pugh A subjects but not in those with Child–Pugh B/C cirrhosis [30]. These discrepant findings may be explained by the lower sample size of Child–Pugh B cirrhosis in our study (50 vs. 149), which hampered detection of potential associations. Moreover, in our study, Child–Pugh C subjects were absent, which generated an underestimation of events in subjects with baseline decompensated cirrhosis.

Our study shows low incidence rates × 100 p/y of liver-related events in SVR cirrhotic subjects. The incidence estimate rate of HCC is 1.6 × 100 p/y; it is similar to the 1.5 rate × 100 p/y reported in the recent prospective Spanish study [13], to the 1.18 rate x 100 p/y reported in the Italian multicenter prospective study by Romano et al., [31] and to the 1.9 rate × 100 p/y reported in a recent retrospective US study [7], confirming the favorable impact of oral antiviral therapy on this severe outcome.

In our study, the incidence rate × 100 person-years of follow-up was 1.7 for liver decompensation events. In particular, 28 patients (4%) developed at least one liver decompensation event (in detail: 16 ascites, 15 variceal bleeding, 5 hepatic encephalopathies, 1 SBP). These data are in line with those of Cheung et al., who showed that liver-related events were most frequent during the treatment period and decreased over time with 16/317 SVR patients (5%) experiencing decompensation in 6–15 months after DAA treatment [15].

The main limitation of this study is the exclusion of Child–Pugh C patients, who, according to the Italian DAAs prescription rules, are not suitable for treatment outside the liver transplantation waiting list. As these subjects are at higher risk of liver-related events, their exclusion may have generated an underestimation of this outcome.

On the other hand, the present study is characterized by several strong points that are worth underlining. First, and most importantly, its prospective design allows for more accurate ascertainment than retrospective studies, which may be affected by selection and ascertainment biases. Second, the large patient cohort, the consecutive enrollment of cases, and the long follow-up (28.4 ± 5 months) represent a unique example in the literature. Third, the cut-off of basal LSM adopted for enrolment (≥14 kPa) allows excluding patients with bridging fibrosis, a subgroup at a lower risk of liver-related events as compared to cirrhotic ones. Finally, the large proportion (86.5%) of subjects who had a coupled liver stiffness measurement at baseline and at the end of follow-up provided enough statistical power to detect the influence of Delta LSM on the occurrence of liver-related events.

## 4. Materials and Methods

### 4.1. Study Design

This was a prospective cohort study. All cirrhotic subjects receiving DAAs therapy were followed for 24 months after confirming SVR (see paragraph SVR). At the end of follow-up, outcome event rates were compared between SVR and no-SVR. Among SVR cases, a further comparison was made among those who developed and those who did not develop liver-related events. In this latter group, adjusted hazard ratios (HR) were calculated to evaluate independent non-serological baseline event predictors (see Statistical Analysis).

### 4.2. Target Population

All consecutive HCV-RNA positive cirrhotic subjects, ≥18 years of age, and Child–Pugh stage A and B candidates for DAAs therapy were enrolled in this prospective study between 1 November 2015 and 31 October 2017 in four tertiary centers (one in Rome, two in Naples, and one in Catania).

The diagnosis of liver cirrhosis was based on the presence of the peculiar clinical, biochemical, ultrasound signs [32], and transient elastometry performed by Fibroscan [16], with a liver stiffness cut-off value ≥ 14 kPa. Exclusion criteria were: history of prior HCC, prior liver transplantation, HBV and/or HIV co-infections, history of alcohol consumption (previous and/or continuing), and Child–Pugh C score since treatment was not reimbursed by the Italian National Health System outside the liver transplantation waiting list [33].

A web database recorded information on baseline demographic, clinical, and treatment characteristics of patients; treatment outcome, type, and timing of event occurrences were also registered.

Subjects signed informed consent to participate. The study was conducted in accordance with the Declaration of Helsinki. The protocol was approved by the ethical board of the promoting center (Federico II University of Naples).

### 4.3. Definition of SVR

SVR was defined as undetectable HCV-RNA at 12 weeks after the end of therapy using the Cobas AmpliPrep/Cobas TaqMan (Roche Molecular Diagnostics, Pleasanton, CA, USA; lower limit of detection 15 IU/mL).

### 4.4. Liver Stiffness Measurement

Liver stiffness measurement (LSM) was performed using transient elastography at baseline and at the end of follow-up by a single experienced operator in each center and according to the usual standard procedure. Improvement in LSM at the end of follow-up (Delta LSM) was defined as a decrease of ≥20% from basal LSM value.

### 4.5. Outcomes and Follow-Up

The main outcome of the study was the analysis of liver-related events development after SVR achievement. Liver-related events were defined as:

- Recurrence or new-onset of 1. ascites, 2. variceal bleeding, 3. spontaneous bacterial peritonitis, 4. hepatic encephalopathy for Child–Pugh A and B patients;
- Occurrence of de novo HCC for Child–Pugh A and B patients;
- In Child–Pugh B patients, worsening of a pre-existing symptom of decompensated cirrhosis (i.e., an increased dose of diuretics, the addition of rifaximin for hepatic encephalopathy, or hospital admission for a new liver failure event).

Any liver-related event that developed during follow-up was registered in the database. Mortality was also registered as liver-related or not liver-related. HCC surveillance was performed with ultrasound every 6 months according to standard clinical practice. In the case of liver focal lesions identified by ultrasound, HCC was confirmed by imaging (computed tomography and/or magnetic resonance imaging) and/or biopsy examination according to international guidelines [34].

The at-risk period for each subject was defined by the time from starting DAAs treatment until the onset of the event. Events occurring before the end of antiviral treatment were not included in the analysis (i.e., 12 subjects: 1 variceal bleeding, 3 ascites, 1 hepatic encephalopathy, 7 HCC). Follow-up lasted 24 months after SVR.

Data were censored when individuals died or were lost during follow-up. Patients lost during follow-up were registered as alive until the last day of observation.

### 4.6. Statistical Analysis

Continuous variables were expressed as mean and standard deviation (SD), and qualitative variables as absolute frequency and percentage. Differences in proportions were evaluated by the Chi-square test; a *p*-value < 0.05 was considered significant.

The relative rate (RR) of liver-related events was calculated as a ratio of the incidence rates of events in the overall cohort of subjects according to the SVR status and in the SVR cohort according to the baseline LSM and its decrease from baseline to the end of follow-up by at least 20%.

The Kaplan–Meier method was used to estimate the cumulative incidence of events. Comparison of survival curves between groups was performed using the log-rank (Mantel–Cox) test.

Incidence density rates of liver-related events per 100 person-years, with 95% confidence intervals (95% CI), were provided.

In SVR patients, univariate and multivariate Cox regression analyses were used to identify baseline variables (age, sex, Child–Pugh class, genotype, and stiffness value) associated with the development of any liver-related event or HCC (outcome variables). Variables with a threshold *p*-value lower than 0.10 at univariate analysis were included in the multivariate model. A sensitivity analysis was performed using Cox proportional hazards regression accounting for the competing risk [35], considering deaths as competing risk events. Assumptions of proportional hazards were tested using Schoenfeld residuals.

The association of baseline characteristics in SVR subjects with an LSM decrement by ≥20% from baseline to the end of follow-up was assessed by a logistic model.

Statistical analyses were performed using STATA 14.2 statistical software (StataCorp, College Station, TX, USA).

## 5. Conclusions

In conclusion, this study identified a basal LSM cut-off of ≥20 kPa as an independent predictor of liver-related events in cirrhotic subjects achieving SVR after DAAs therapy. Studies with a longer follow-up in this setting are warranted to further evaluate the potential role of Delta LSM on the occurrence of liver-related events.

## Figures and Tables

**Table 1:** Baseline characteristics of cirrhotic subjects who attained SVR and those who did not after treatment with DAAs.

Baseline Characteristics		Total Subjects (*n* = 706)	SVR (*n* = 687)	Non-SVR (*n* = 19)	
			*n* (%)	*n* (%)	*p*
Sex	Males	387 (54.8)	374 (54.4)	13 (68.4)	0.227
	Females	319 (45.2)	313 (45.6)	6 (31.6)	
Sex ratio	M/F	1.2	1.2	2.2	
Age Distribution (years)	≤50	101 (14.3)	98 (14.3)	3 (15.8)	
	51–65	239 (33.9)	236 (34.3)	3 (15.8)	0.229
	>65	366 (51.8)	353 (51.4)	13 (68.4)	
Mean age (SD)		64.1 (11.7)	64.0 (11.7)	64.9 (11.8)	0.752
Previous IFN treatment		379 (53.7)	367 (53.4)	12 (63.2)	0.401
Child–Pugh *	A	651 (92.2)	637(92.7)	14 (73.7)	0.002
	B	55 (7.8)	50(7.3)	5 (26.3)	
Mean Child–Pugh score (SD)		5.3 (0.7)	5.3 (0.7)	5.7 (1.0)	0.008
Stiffness (kPa) **	<20	411 (70.9)	400 (70.6)	11 (84.6)	
	≥20	169 (29.1)	167 (29.4)	2 (15.4)	0.253
Mean liver stiffness		19.2 (7.9)	19.3 (8.0)	16.7 (3.3)	0.253
Genotype	1	567 (80.3)	550(80.1)	17 (89.5)	
	2	96 (13.6)	95(13.8)	1 (5.3)	
	3	27 (3.8)	27(3.9)	0	0.578
	4	15 (2.1)	14(2.0)	1 (5.3)	
	other	1 (0.1)	1(0.2)	0	
DAAs Therapy	SOF	60 (8.5)	58 (8.4)	2 (10.5)	
	SOF + other	460 (65.1)	446 (65.0)	14 (73.7)	0.697
	3D	160 (22.7)	157 (22.8)	3 (15.8)	
	2D	26 (3.7)	26 (3.8)	0	
Ribavirin		316 (44.8)	307 (44.7)	9 (47.4)	0.817

* None of the subjects were Child–Pugh C; ** Stiffness measurements performed in 567 (82.5%) subjects.

**Table 2:** Number and incidence rate per 100 person-years of main events during the 24 months of follow-up after the end of treatment with DAAs in patients who attained SVR.

Events	*N*	Incidence Rate Per 100 p/y
Overall death	20	1.2 (0.8–1.8)
Death due to hepatic cause	8	0.5 (0.2–1.0)
Death due to non-hepatic cause	12	0.7 (0.4–1.3)
Any liver decompensation *	28	1.7 (1.2–2.4)
HCC ^#^	26	1.6 (1.1–2.3)
Liver transplantation	1	0.1 (0.0–0.4)
Any event ^§^	61	3.7 (2.9–4.8)

* Ascites, hepatic encephalopathy, upper gastrointestinal bleeding, SPB (spontaneous bacterial peritonitis); # five subjects also developed liver decompensation; ^§^ includes all the events listed in the table.

**Table 3:** Frequency of baseline characteristics of SVR subjects according to a ≥20% decrease in liver stiffness value after 24 months of follow-up.

Characteristics		Decreased Stiffness		*p*-Value *
		Yes (*n* = 258)	No (*n* = 232)	
		*n* (%)	*n* (%)	
Sex	M	140 (54.3)	117 (50.4)	0.396
	F	118 (45.7)	115 (49.6)	
Age (years)	<65	124 (48.1)	118 (50.9)	0.536
	≥65	134 (51.9)	114 (49.1)	
Child–Pugh	A	250 (96.9)	225 (97.0)	0.957
	B	8 (3.1)	7 (3.0)	
Genotype	1	200 (77.5)	197 (84.9)	0.037
	other	58 (22.5)	35 (15.1)	

* Chi-squared test. Limited to subjects who had stiffness measurement at baseline and at the end of follow-up. Subjects who died due to a hepatic or non-hepatic cause and who underwent liver transplantation were excluded.

**Table 4:** Crude and adjusted hazard ratios (HR) for the association of baseline characteristics with liver decompensation and HCC in cirrhotic subjects who attained SVR after DAAs therapy (univariate and multivariate Cox model).

Characteristics		Liver Decompensation				HCC			
		HR_crude_ (95% IC)	*p*	HR_adj_ (95% IC)	*p*	HR_crude_ (95% IC)	*p*	HR_adj_ (95% IC)	*p*
Sex	F	1		-		1		*-*	
	M	1.0 (0.5–2.2)	0.931	-		2.1 (0.9–5.2)	0.080	1.6 (0.5–5.2)	0.436
Age (y)	<65	1		1		1			
	≥65	2.6 (1.1–6.2)	0.030	3.2 (0.8–12.0)	0.091	1.6 (0.7–3.6)	0.248	*-*	
Child–Pugh	A			1					
	B	8.0 (3.5–18.2)	<0.001	3.8 (0.4–34.4)	0.236	2.6 (0.9–7.7)	0.079	2.8 (0.6–12.8)	0.191
Stiffness	<20	1		1		1			
	≥20	3.8 (1.1–13.4)	0.034	4.0 (1.1–14.1)	0.031	8.3 (2.3–30.1)	0.001	7.2 (1.9–26.7)	0.003
Genotype	1	1		1		1			
	other	2.6 (1.2-5.6)	0.020	7.5 (2.1-27.3)	0.002	1.7 (0.7-4.0)	0.253	*-*	
DAAs therapy	SOF-based	1		-		1		*-*	
	other	0.5 (0.2-1.5)	0.213	-		0.6 (0.2-1.6)	0.326		

## Data Availability

No new data were created or analyzed in this study. Data sharing is not applicable to this article.

## Supplementary Materials

The following are available online at https://www.mdpi.com/article/10.3390/cancers13153810/s1: Table S1. Incidence rate (IR) per 100 person-years and relative rate (RR) of liver-related events in SVR vs. no SVR cases (A), of liver decompensation and HCC in SVR according to Delta LSM (B), and basal LSM value (C) in an Italian prospective study.
