# Supplementary material for: Risk Factors for Liver Decompensation and HCC in HCV-Cirrhotic Patients after DAAs: A Multicenter Prospective Study
Source: Cancers (Basel). 2021 Jul 29;13(15):3810. doi: 10.3390/cancers13153810 (PMC8345116; doi:10.3390/cancers13153810)
Supplement: Supplementary file 1 [file cancers-13-03810-s001.zip › cancers-1249965-supplementary.pdf]

cancers

MDPI

Risk Factors for Liver Decompensation and HCC in HCV-Cirrhotic Patients after DAAs: A Multicenter Prospective Study

Filomena Morisco, Alessandro Federico, Massimo Marignani, Mariarita Cannavò, Giuseppina Pontillo, Maria Guarino, Marcello Dallio, Paola Begini, Rosa G. Benigno, Flavia L. Lombardo and Tommaso Stroffolini

Table S1. Incidence rate (IR) per 100 person-years and relative rate (RR) of adverse events in SVR vs. no SVR cases (A), of Liver Decompensation and HCC in SVR according to Delta LSM (B) and Basal LSM value (C) in an Italian prospective study.

**Characteristic**	**Any Event IR (95% CI)**	**Any Event RR (95% CI)**	**Liver related death IR (95% CI)**	**Liver related death RR (95% CI)**
**A**				
**SVR Yes**	**3.7 (2.9-4.8)**	**3.3 (1.3-8.2)**	**0.5 (0.2-1.0)**	**9.0 (1.9-42.6)**
**SVR No**	**12.3 (51-29.6)**		**4.3 (1.1-17.2)**	

**Characteristic**	**Liver Decompensation IR (95% CI)**	**Liver Decompensation RR (95% CI)**	**HCC IR (95% CI)**	**HCC RR (95% CI)**
**B**				
**Delta LSM**				
**≥ 20%**	1.2 (0.3-5.4)	0.5 (0.2-1.6)	0.3 (0.1-1.4)	2.3 (0.4-11.8)
**< 20%**		0.6 (0.3-1.7)	0.8 (0.3-1.9)	
**C**				
**Basal LSM value**				
**< 20 kPa**	5.5 (1.7-17.8	0.4 (0.1-1.1)	0.3 (0.1-0.9)	8.3 (2.3-30.2)
**≥ 20 kPa**		2.2 (1.2-4.3)	2.5 (1.4-4.7)	
